# A large-scale mass casualty simulation to develop the non-technical skills medical students require for collaborative teamwork

**DOI:** 10.1186/s12909-016-0588-2

**Published:** 2016-03-08

**Authors:** Christine Jorm, Chris Roberts, Renee Lim, Josephine Roper, Clare Skinner, Jeremy Robertson, Stacey Gentilcore, Adam Osomanski

**Affiliations:** Sydney Medical School, University of Sydney, Sydney, 2006 NSW Australia; Lend Lease, Sydney, 2000 NSW Australia; Hornsby Kuringai Hospital, Sydney, 2077 NSW Australia

**Keywords:** Simulation, Acceptability, Fidelity, Reflection, Collaboration, Professionalism, Large group teaching, Non-technical skills, Teamwork, Mass casualty, Patient safety

## Abstract

**Background:**

There is little research on large-scale complex health care simulations designed to facilitate student learning of non-technical skills in a team-working environment. We evaluated the acceptability and effectiveness of a novel natural disaster simulation that enabled medical students to demonstrate their achievement of the non-technical skills of collaboration, negotiation and communication.

**Methods:**

In a mixed methods approach, survey data were available from 117 students and a thematic analysis undertaken of both student qualitative comments and tutor observer participation data.

**Results:**

Ninety three per cent of students found the activity engaging for their learning. Three themes emerged from the qualitative data: the impact of fidelity on student learning, reflexivity on the importance of non-technical skills in clinical care, and opportunities for collaborative teamwork. Physical fidelity was sufficient for good levels of student engagement, as was sociological fidelity. We demonstrated the effectiveness of the simulation in allowing students to reflect upon and evidence their acquisition of skills in collaboration, negotiation and communication, as well as situational awareness and attending to their emotions. Students readily identified emerging learning opportunities though critical reflection. The scenarios challenged students to work together collaboratively to solve clinical problems, using a range of resources including interacting with clinical experts.

**Conclusions:**

A large class teaching activity, framed as a simulation of a natural disaster is an acceptable and effective activity for medical students to develop the non-technical skills of collaboration, negotiation and communication, which are essential to team working. The design could be of value in medical schools in disaster prone areas, including within low resource countries, and as a feasible intervention for learning the non-technical skills that are needed for patient safety.

## Background

When healthcare teamwork and communication are improved, medical errors are fewer and the quality of care better [1, 2]. Despite its strengths, work-based learning in the clinical space is ‘messy, unpredictable, unregulated, fragmented and under-theorized [3]’, and medical students may not experience being an integral healthcare team member. They may witness teamwork or its absence, but without reflection, personal learning may be limited.

Teamwork is central to the acquisition of non-technical skills (NTS), which have been described as ‘the cognitive, social and personal resource skills that complement technical skills, and contribute to safe and efficient task performance [4]’. They are believed to be a crucial component of preparation for practice [5].

While simulation suites are now commonplace, particularly in medium to high resource countries, many medical students receive limited simulation experiences to develop their non-technical skills. Educators often believe that the authenticity and fidelity of simulation need to reflect the complexity of the skill being learned [6] and that high fidelity is necessary for teaching complex skills like teamwork [7]. Designs that are resource and time intensive are not sustainable for delivery to large cohorts. Yet there is little evidence on the scale at which simulation can be successful. At the same time, it is known that interactive large group teaching can produce good learning outcomes [8]. Studies comparing high and low fidelity simulations have shown minimal increases in learning, as long as a ‘baseline authenticity’ is obtained [9].

Fidelity exists in two major planes; ‘engineering’ (physical) and psychological, the latter being more powerful in assuring effective learning, once essential critical elements exist in the scenario [9]. Despite engineering triumphs such as computerised voices and bleeding wounds, simulations frequently lack ‘sociological fidelity [10]’. This form of fidelity takes account of the integration of a complicated range of skills, attitudes and behaviours, which require a firm understanding as to how factors such as imbalances of authority and influence, impact on collaborating and negotiating processes (these are also complex technical activities in their own right).

An opportunity to investigate some of these issues arose in the design and implementation of a large class learning and teaching activity, framed as a simulation of a natural disaster, in order for medical students to develop the non-technical skills essential to health care team working.

### Educational design

The mass casualty scenario was designed to take account of the research evidence on effective ways to encourage students to work in teams and acquire non-technical skills [11], in particular their collaboration, negotiation and communications skills. Secondary learning outcomes for the students included the demonstration of situational awareness, clinical risk assessment and prioritisation of the clinical cases. Our design was creative because there is little existing literature on student centred large-scale simulations. Large student groups have watched complex live simulations [12] and small groups of nursing students have practiced formal disaster triage with a set-up similar to ours (i.e. focus on psychological fidelity) [13], and some very large health system exercises have been designed to test emergency service responses [14].

The students were in their second year of a 4-year graduate entry problem-based medical program and would be challenged by the complex clinical problem solving scenarios, in a psychologically safe but relatively stressful learning environment. Overarching learning objectives around teamwork were assessable, as part of the vertical personal and professional development curricular theme and thus the activity was supported by relevant lectures and reading available in the on-line Learning Management System. The educational design was suitable for the full cohort (*n* = 320 students) but attendance was voluntary, as the activity was designed as pilot to evaluate the feasibility of embedding the activity within the curriculum.

We simulated an earthquake resulting in the collapse of a church near a medical student convention venue in New Zealand, deemed highly appropriate given recent earthquake activity in the Asia Pacific Region. An earthquake creates enough destruction and damage to require many helpers, allowing medical students to act in their role, here as first responders. This differs from many simulations where the student is required to imagine being a junior doctor. Attempts by simulators at physical fidelity can distract from psychological [9] and sociological fidelity. In order to achieve ‘learner engagement and suspension of disbelief [7]’ , we focused on techniques such as soundscapes (sirens, loud earthquake video footage) and upturned chairs to give the appearance of structural environmental damage.

**Fig. 1:**
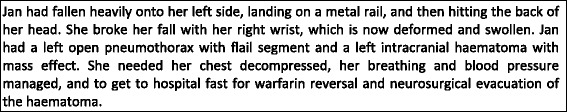
Example simulated patient details for one of the four simulated earthquake casualties used in the simulation

Students were assigned to work in groups of four or five, and attend one of four simulated clinical cases developed by experienced emergency and pre-hospital resuscitation physicians. These cases (hair stylist Jan, university student Tom, 34 weeks pregnant Gloria and retired public servant Harold) were designed to be realistic, including a ‘real time’ aspect to changes in their condition. The detail of one of these simulated patients is given in Fig. 1. The clinical cases differed from each other (e.g. in only one was CPR required), however, the storylines were similar and designed to deliver specific learning outcomes [15]. They were adjusted so that each had a staged progression. Each student team thus faced a similar range of teamwork challenges while caring for their patient, e.g. a first aid stage while the team roles formed (or not), the introduction of uncertainty when patient deterioration occurred, and options to delegate and negotiate for limited treatment and diagnosis resources.

One student in each group was designated to act as the simulated patient. All patients were instructed to be mute (unconscious or in severe pain or short of breath) in order to limit the need for student acting and potential inconsistencies across cases.

**Fig. 2:**
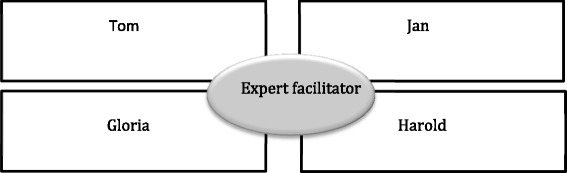
Structure of one of eight ‘disaster clusters’ comprising four simulated patients, with 1 student simulating the patient and attended by 3–4 students

The large hall venue was divided into eight ‘disaster clusters’ (see Fig. 2) containing one instance of each of the four simulated patients. An expert educator (an emergency medicine or retrieval physician or nurse consultant with simulation expertise) facilitated each cluster. Thus each facilitator supported 12–16 students each.

**Fig. 3:**
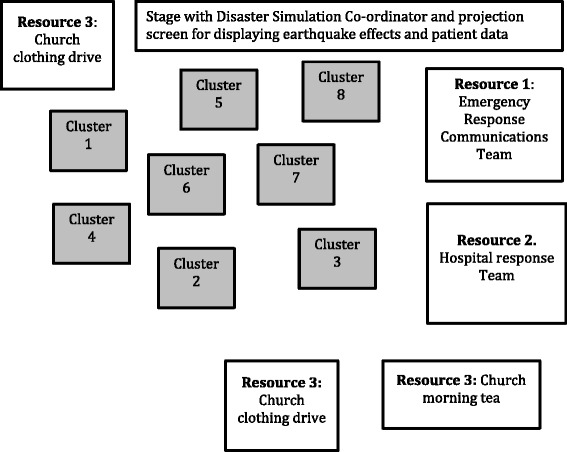
Mass trauma simulation design, demonstrating eight disaster clusters, each containing one facilitator, four simulated patients, from 12 to 16 attending students, and three differing types of resources to support student team working

There were three types of resources that students could access during the simulation. First, the Emergency Services Communications team provided students with information about survivors including the simulated patients, in order to assist them during the simulation. This was achieved by using the university text messaging system, normally used to relay information to students’ mobile phones, about timetabling issues. Second, the Hospital Response Team provided specific clinical advice and provided simulated medical treatment and equipment, for example intravenous fluids, injectable analgesia and access to a field x-ray machine. Third, the ’church clothing drive’ contained sheets, blankets and old clothing for warmth and the ‘church morning tea’ offered cold drinks and biscuits for hydration and nutrition, and cling wrap for wound care. The overall design of the venue is given in Fig. 3.

**Fig. 4:**
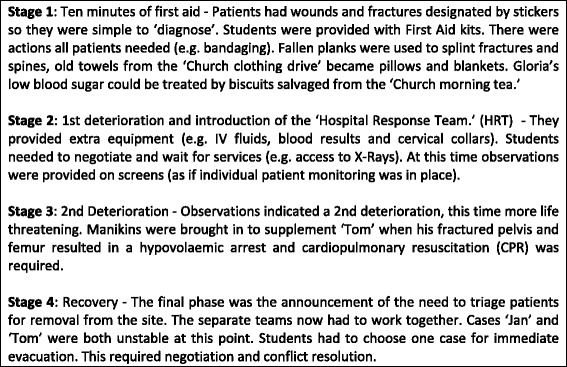
Outline of staged case progression over the course of the simulation

After 15 min of orientation to the exercise by the overall simulation co-ordinator from the stage, earthquake footage and sirens announced the start of the simulation. The students were asked to be ‘first responders’. Each case was progressed in four stages over a period of 50 min and directed from the simulation co-ordinator’s area. Patient observations were made available for Stages 2–3, via the large screen on the stage. A scaffolded approach was used [16] with students given an initial opportunity to practice successfully in Stage 1. In Stage 2, the ‘Hospital Response Team (HRT) staffed by Faculty provided extra equipment and advice. By Stage 3, students were well established in the scenario structure and could focus on more complex medical issues and team function. The students’ actions made no difference to the patients’ vital signs. The patients rallied and deteriorated – just as in the real world, good care was not necessarily rewarded by patient improvement or survival (Fig. 4).

A number of realistic distractors were used in order to promote authenticity and fidelity, given that mass trauma due to a disaster will be a chaotic situation. As an example, a professional actor playing an uninjured character sought attention from the students, as they were caring for their patients. The text messaging service was utilised to send patient information, which included irrelevant distractors and requests for the students to leave their patients to relay information to the ‘Emergency Services Communications team’. For instance, Jan’s daughter needed to know if she should get on a flight from the UK to New Zealand.

At the end of the simulation, the disaster cluster facilitators conducted a 30 min formal debrief with their groups of students. Debriefing is critical to the achievement of student learning outcomes, as ‘the potential of reflection for individuals may not be fully realized without the help and support of another person’ [17] or a formal process [18, 19]. The experience of challenging teamwork was investigated, with particular attention on the learning outcomes of collaboration, negotiation and communication skills, but also the secondary outcomes of situational awareness. At the end of the activity, microphones were then provided and each group’s learning and facilitator observations about their experience with their case were shared with the whole class.

#### Staff orientation and preparation

All staff received a detailed written scenario schedule in advance of the simulation and participated in a 30-min briefing prior to students’ arrival. Staff were briefed to encourage student decision-making and assumption of responsibility, and the Emergency Services Communications team was encouraged to be realistically pragmatic, for example sending students back to their groups for extra information. Cluster facilitators received full patient details in advance. Staff who were assigned to the Hospital Response Team were familiar with the equipment and services they offered, but only learnt the details of patients as the simulation played and students made requests for supplies. Thus the Hospital Response Team staff became actively involved with the students in communication and negotiation and prioritising use of resources.

Our research aim was to determine the acceptability and effectiveness of a simulated mass casualty emergency situation designed to develop medical students’ non-technical skills of collaboration, negotiation and communication.

## Methods

Our theoretical lens for determining how effectively students achieved the learning outcomes for the simulation were based on the five key concepts of Schön’s model of the reflective practitioner: knowing-in-action, surprise, reflection-in-action, experimentation and reflection-on-action [20]. We anticipated that students as learners could demonstrate what they knew initially through largely intuitive actions or, to paraphrase Schön, “their knowing is their action”. When a student encounters something they have never seen before, Schön called this a “surprise” ([21] p.26). The student is anticipated to “reflect-in-action” during the first encounter, find a “surprise”, go and get more information, then bring that information back to the problem and then experiment. The student then “reflects-on-action” afterwards and adds to their knowledge base ([21] p.26). Multiple surprise elements were designed to challenge students to develop and maintain technical skills and the non-technical skills inherent in teamwork. The model of the reflective practitioner is used extensively in the literature around debriefing following simulation [19], and more broadly in emergency medicine, and is inclusive of attending to emotions [22]. The demonstration of reflection-on-action [21] would equate to level two on Kirkpatrick’s four level model of evaluating training programs [23] demonstrating the extent to which participants acquire the intended knowledge, skills, attitudes, confidence and commitment based on their participation in a training event.

### Ethics

The Sydney Medical Program has standing approval from the Chair of the Human Research Ethics Committee of the University of Sydney on 4 February 2010 to conduct analyses of de-identified assessment and admission data that are for quality improvement purposes. Our study fulfilled these requirements.

### Data collection

This research was part of a larger mixed methods study [24] evaluating the feasibility of the activity for the whole class. Observational field notes of the students in their clusters [25] were made by six of the authors who had participated in the simulation and debriefing (RL, CJ, JR, JR, AO, SG) and written up after the event, to capture their reflections. These observations included the perceptions of the students assigned to be patients about their management and observations of teamwork. In the broader health professions education literature, observation participation methods [26] have both shaped and generated detailed understandings of different topics e.g. interprofessional learning [27] and in postgraduate medical education settings, for example, to explore the acquisition of knowledge in anaesthetic practice [28]. At the end of the activity, students were given the opportunity to complete an anonymous short teaching evaluation questionnaire containing checklist items with a 5-point Likert scale, which provided opportunity for open-ended comments at the end of the activity. The checklist items explored the degree of their engagement in the learning, their perceptions of the value of the activity to their learning, and the educational impact. Free text comments were invited on what worked well in the simulation, what didn’t work so well, and suggestions for improvement.

### Data analysis

The evaluation questionnaire was analysed using SPSS Version 19 [SPSS Inc., Chicago, IL, USA] and we report the acceptability measures here. Qualitative comments made by 99 %, (116/117) of participating students and observer participation records were collated and analysed as a data set [29] by two of the authors CR and CJ, both experienced qualitative researchers in medical education and health services research. Thematic analysis is a method for identifying, analysing and reporting patterns (themes) within the data that are important to the description of a phenomenon and are associated with our specific research question. Thematic analysis was performed through the process of coding in six phases: familiarization with data, generating initial codes, searching for themes among codes, reviewing themes, defining and naming themes and producing the final report [24]. Two authors, (CJ and CR) firstly immersed themselves in the data, and working inductively, generated a series of initial codes, which were then clustered into themes. The authors negotiated meanings by moving iteratively between the data and the research question in the context of our theoretical lens and negotiating meaning where there was disagreement. The coding framework was presented to all the authors for their critical appraisal [29]. Student quotes are identified by their identifying number in the dataset and by the initials of staff observer participants.

## Results

There was a 100 % response rate to the checklist items on the questionnaire (*n* = 117) with 69 % rating the activity as very engaging, and 34 % rating it as engaging. Similarly 94 % thought it very worthwhile or worthwhile, 95 % thought it to be very memorable or memorable, demonstrating acceptability (see Table 1).

**Table 1:** Quantitative student evaluation results showing students acceptability of the simulation as a learning activity. (*n* =117)

Overall the simulation was				
Very engaging	Engaging	OK	Boring	Very Boring
68 (59 %)	39 (34 %)	5 (4 %)	0	0
This was a worthwhile activity				
Strongly agree	Agree	Neutral	Disagree	Strongly Disagree
72 (63 %)	36 (31 %)	6 (5 %)	0	1 (1 %)
Some learning will be memorable for me				
Strongly agree	Agree	Neutral	Disagree	Strongly Disagree
67 (58 %)	42 (37 %)	5 (4 %)	0	1 (1 %)

The qualitative data supported three themes in relation to answering our research question about the educational effectiveness of the activity. First, the relationship between the fidelity of our large class mass casualty simulation and student engagement with learning. Second, reflexivity which included students attending to emotions, and students providing insight into their own learning. And third, the extent to which the exercise successfully led to learning about non-technical skills around collaboration, negotiation and communication.

### The impact of fidelity on learning

The novelty of the exercise in the early years of a medical course appealed, with one student noting the innovation in contrast with more traditional learning and assessment methods,

‘Being put in a situation where fast thinking and skills needed, not just multiple choice questions’. (S54)

The fidelity of the simulation including the simulated patients was largely acceptable, with relatively simple enhancements suggested, for example ‘the realism was fantastic - could maybe add smoke machines’ (S51). Suggestions for improvement also included potentially more resource intensive and higher fidelity options such as professional actor patients and patient vital signs and observations that were responsive to student actions. Students valued the experience of ‘dealing with real life patients’ (S97), and noted ‘how chaotic and unorganised the emergency situation was’. (S8) They relished the chance within a safe setting to undertake,

‘the management and decision making in an acutely stressful situation where life and death outcomes prevail’. (S58)

For some students the creation of an authentic simulated learning environment allowed new learning.

‘Learning how to learn something new and put it into practice within an acute setting’. (S105)

Some requested further opportunity to consolidate new learning by having an additional scenario to apply their newly acquired skills. One student suggested ‘Multiple or at least 2 scenarios so people could act on things learnt in the 1st scenario’. (S26)

### Reflexivity

The rich reflective opportunities that the mass trauma simulation created were evident within the examples of reflection-in-action and reflection-on-action being volunteered by students. While the focus of the learning activity was on non-technical skills, students were also able to reflect on the importance of basic technical skills despite being inundated with rich contextual information, which demanded the application of negotiation and communication skills.

‘Needing to know what to do, it was overwhelming being faced with a situation where you don’t know but I was surprised how the basics came in. Assessing vitals and taking a history. It’s helped me feel more confident’. (S29)

While this student was able to recognise their ‘knowing-in-action’ [20], others realised the rich contextual information in the simulation had made them forget the basics, for example one student said she had ‘tunnel vision, realising the questions I didn’t even think to ask’. (S94)

Our data suggested important learning was gained from the students’ reflection on their behaviour and their critical thinking skills during the simulation: ‘I realised that loss of information occurs in stressful situations’. (S95) Some got to understand their own behaviours, and reflect on how they might behave in future. For example the extrovert who suggested: ‘I dominate too much sometimes’ (S55) and the introvert who noted the need to ‘Speak out and be heard!’(S86).

One of the students referred to a moment when a manikin was introduced into the scenario when ‘Tom’, one of the simulated patients required CPR. A student who had been looking after ‘Tom’ said ‘I can’t believe how involved I got, I just really wanted him to survive’. (CJ -Field Note)

Students acting as patients, described feelings of fear, worry, and helplessness suggesting sufficient fidelity was present for immersive engagement. However, the facilitators made several field notes about the care and tenderness students displayed for their ‘patients’. As well as verbal reassurance, students were observed physically cradling and supporting the injured. Emotional engagement was also suggested by the student who remarked on the experience of having ‘Limited resources and having to *fight* for them for *your* patient in an emergency’. (S82)

However, not all students found the learning experience valuable to their learning, requesting ‘More assistance when we're flailing’ (S63) or suggesting

‘It was really good but needs to be followed up with teaching as opposed to just being a one-off demonstration of our incompetence’. (S6)

### Collaborative team working

The simulation was intended to develop collaboration, negotiation and communication skills. Some students recognized the importance of leadership, in both negotiating and communicating about their patient with each other and with the Emergency Services Communications and Hospital Response Teams.

‘Making sure to check what’s going on in the surrounding area, having someone take charge’. (S18)

However a surprising number of the groups failed to decide on team structure and process. Reasons for this were explored in the debriefing and included the novel pressured environment and the students’ desire to act quickly to help their patients. By participating in teamwork students learnt the importance of ‘prioritising injuries and delegating’ (S68) and ‘constant communication—debrief team every time situation changes’ (S40). Many students discussed how they reflected during the simulation on difficulties they were having and experimented with differing roles and structures:

‘That structure helps - as soon as a team leader is appointed, and you followed systems, that’s when things worked. Writing things down is also crucial (for your own sake and others)’. (S44)

Not all student groups were able to focus on the non-technical skills, instead focussing on technical clinical issues that excluded the patient. Students who acted as patients found much to reflect on the actions of others. For example:

‘…it was a rare opportunity for me to be on the other side of health care. It was often very disorienting and at times worrying as I could hear everything around me but was helpless to do anything’. (S106)

Another observed a lack of decision-making:

‘I was the patient and felt frustrated at how difficult it was for everyone to decide what to do’. (S49)

The surprises and resource constraints built into the scenario were valued and indicated that the exercise was effective, for at least some, in bringing attention to situational awareness, one of the secondary learning outcomes

‘Learning how you react to a situation and how difficult it is to find things as well as gather info from the patient’. (S114)

## Discussion

Our data indicated the acceptability and effectiveness of a large class simulation of natural disaster in demonstrating non-technical of skills of collaboration, negotiation and communication as learning outcomes. All students participated in the teamwork learning experience simultaneously [15] most finding it an acceptable way to learn. We provided evidence of the effectiveness of the scenario in three ways. First, physical fidelity was sufficient for good levels of student engagement, however for most students there was also sociological fidelity [10]. The fidelity was sufficiently fit for purpose, showing that the low fidelity simulation provided a reasonable match to the students’ learning needs [30]. Second, students were able to both reflect-in-action, during the learning activity and reflect-on-action at the debriefing about what they had learned. Third, students developed and demonstrated their acquisition of the important non-technical skills through their willingness to collaborate, negotiate and communicate, rather than reacting negatively or defensively to such circumstances [31]. Situations and problems for which solutions were unclear or suboptimal arose, and the students’ reflections helped them make sense of these situations and allowed them to function in the “messiness” of an emergency situation [22]. The acceptability and effectiveness of this simulation activity, which was deliberately low fidelity, should prove attractive to medical schools in low resource countries. It might be particularly valuable in areas of the world where there are a high incidence of natural disasters.

There are a number of implications for medical and health educators wishing to develop large scale disaster simulations to provide teaching and learning around team skills. Effectiveness research aims to identify educational interventions that can work in a variety of settings [32]. In particular understanding how refinements to the educational design can be made, and costs managed so health educators may be able to adapt this model for success with their own students.

While there are highly protocolised emergency responses able to be successfully rehearsed by teams in high fidelity simulation settings, most health care work is not routine [33] and often chaotic. Our educational design focussed on constructing a learning environment that necessitated experimentation and reflection. It provided unexpected challenges that allowed students to be creative about ambiguity and surprise, rather than reacting negatively or defensively to such circumstances [31]. However, there is also an argument, well founded in cognitive psychology that rich contextual information (distractions) in simulation makes performing a skill and learning more difficult [34]. Our provision of these elements was not extraneous but, as our data indicate, resulted in learner engagement. For some students simulation resulted in transformational learning about teamwork in a safe space which reflected the uncertainties of clinical practice and recreated the conditions of real-world learning [35]. Much student teaching and learning using simulation is highly structured and thus risks ‘an insulated simulated context learning experience’ with exercises isolated from the nature of work [3]. Whilst practiced clinical routines (the ‘ABCs’) enable novices to make an immediate start on clinical problems, challenges such as our activity, require students to identify their needs for future non-technical skill development. A simple scenario does not allow students to learn that: ‘To act effectively in complex situations, team members need to pay attention to all possible sources of information, and integrate that information into team action’ [36].

If one of the goals of medical simulation is to have learners understand and confront their limitations [37], our students learnt for instance, that to communicate with others in crisis situations is paramount. In the interests of patient safety, educators have recently been challenged ‘to seek ways in which to expose students to opportunities for medical error, whilst developing safe-guards that adequately protect patients’ [38]. Experience is necessary to enable students to ‘develop acceptance of their own fallibility’ [38] and develop their ability to attend to emotions that being in the clinical work environment requires. Large scale challenging simulations can provide such experiences for students. Reflective engagement in challenging teamwork scenarios should give students more confidence in managing uncertainty—whether in daily ward work or in the context of disaster.

Internationally, attention is being paid to cost effectiveness in evaluations of medical education and the costs of exercises in high fidelity simulation suites, while poorly reported [30], are high. Disaster medical education itself is a new and evolving field and experts have an interest in the development of: ‘low cost, scalable training approaches’ [39] that help preparation for low frequency events. The improvisation we required around resources is a classic element in disaster simulations [40]. However in this exercise, we did not focus on the management of the casualties as a whole. Where resources are available, to enhance preparedness for real life situations, an educational event such as the one we have described could be followed by ‘real-time, team-in-the-loop’ [39] virtual disaster simulation so that students could be exposed to command and control strategies. Whilst we did not do a formal cost benefit exercise, there were no additional costs for equipment, and everything we used would be reproducible in low resource health education settings. Further research is required to determine whether a low-fidelity simulation can provide achievement of a range of learning outcomes when fully embedded in the curriculum [30]. It could investigate whether future large-scale events could be run with fewer tutors, and further reducing costs without losing educational effectiveness [30].

### Strengths and limitations of the study

To our knowledge this is the first student-based large-scale simulation of a natural disaster to be reported. We acknowledge the debate around abandoning the use of the term fidelity [7, 34], replacing it with terms reflecting the underlying primary concepts of physical resemblance and functional task alignment, we use it here for clarity in describing our design and its rationale in terms used by existing literature. A dilemma in observer participation studies in educational settings, is balancing the importance of description of the phenomenon under study versus interpretation. That is understanding the perspectives of the students being studied and developing an analytic understanding of their perspectives, activities and actions [26]. We acknowledge that there are limitations in generalising the findings of a qualitative study to other settings.

## Conclusion

We have demonstrated the acceptability and educational effectiveness of a large-class teaching model for students learning the non-technical skills of collaboration, negotiation and communication in a simulated disaster medicine context. Our findings will be of interest to medical schools in disaster prone areas, including within low resource countries, and as a feasible intervention for learning the non-technical skills that are needed for patient safety.
